# Decoy receptor 3 as a diagnostic marker for sepsis: a meta-analysis

**DOI:** 10.1186/s40635-026-00943-z

**Published:** 2026-07-02

**Authors:** Xiao-Ke Zhang, Rong-Yue Gao, Yu-Zhen Han, Shu-Ya Hou, Wen-Xiong Li, Li-Feng Huang

**Affiliations:** Department of Intensive Care Unit, Beijing Chao-yang Hospital, Capital Medical University, 8 Gongren Tiyuchang Nanlu, Chaoyang District, Beijing, 100020 People’s Republic of China

**Keywords:** DcR3, Sepsis, Diagnosis, Biomarker

## Abstract

**Objective:**

Sepsis is a syndrome of systemic inflammatory reaction caused by a severe infection, leading to multiorgan damage and a high mortality rate. In recent years, several studies have shown that decoy receptor 3 (DcR3) is positively correlated with the severity of infection, and its high specificity and sensitivity in the diagnosis of sepsis is expected to serve as a novel marker for sepsis. This meta-analysis aims to evaluate the diagnostic accuracy of decoy receptor 3 (DcR3) for sepsis in the intensive care unit (ICU) setting.

**Methods:**

We conducted a comprehensive search in six databases, extracting data independently.Studies were included if they assessed the diagnostic accuracy of DcR3 for sepsis in the intensive care unit (ICU). A meta-analysis was performed using a random-effects model to calculate pooled sensitivity, specificity, and area under the curve (AUC).

**Results:**

Four records assessing 681 patients were included in this meta-analysis. In distinguishing sepsis from normal controls, DcR3 demonstrated exceptional discriminatory power with an AUC of 0.99. The pooled sensitivity was 0.98 (95% CI 0.95–0.99), and the pooled specificity was 0.95 (95% CI 0.92–0.97). The pooled PLR was 21.21 (95% CI 12.71–35.40), the pooled NLR was 0.02 (95% CI 0.01–0.06), and the pooled DOR was 878.26 (95% CI 217.36–21062.55). In differentiating sepsis from systemic inflammatory response syndrome (SIRS), the AUC was 0.95. The pooled sensitivity was 0.93 (95% CI 0.87–0.96), and the pooled specificity was 0.87 (95% CI 0.68–0.95). The pooled PLR was 6.89 (95% CI 2.67–18.26), the pooled NLR was 0.08 (95% CI 0.04–0.15), and the pooled DOR was 86.17 (95% CI 27.35–271.55).

**Conclusion:**

On the basis of our meta-analysis, DcR3 is a helpful marker for early diagnosis of sepsis on ICU admission. However, the findings are limited by the small number of included studies and significant heterogeneity. Further large-scale, multicenter prospective studies are warranted to validate these results.

**Supplementary Information:**

The online version contains supplementary material available at 10.1186/s40635-026-00943-z.

## Background

According to the Sepsis 3.0 diagnostic criteria, sepsis is defined as a life-threatening organ dysfunction caused by a dysregulated host response to infection [1]. Sepsis is a common complication of severe trauma [2], burns [3], infections, major surgeries [4], immunosuppression, and advanced tumors [5], and has long been a major admission to intensive care unit (ICU) wards. When the inflammatory response triggered by infection is out of control, multi-organ damage and septic shock will develop rapidly, which has the characteristics of high incidence, rapid progress, poor prognosis, high mortality rate, and high cost of treatment. Epidemiological studies show that in 2017, there were about 48.9 million people suffered from sepsis globally, and 11 million deaths from sepsis-related cases, which accounted for 19.7% of the total number of deaths globally [6]. The latest data show that 19 million new cases of sepsis occur globally each year, with a mortality rate higher than that of stroke, which undoubtedly imposes a heavy burden on public health [7]. Thus, rapid detection of sepsis is crucial for timely treatment, prevention of adverse outcomes, and reduction of mortality.

Inflammation-mediated cytokine storm, apoptosis-induced lymphopenia, and prolonged immune paralysis constitute the three typical immune features of sepsis, and given the complex pathophysiology of sepsis and the individual differences of patients, it is important to comprehensively consider the clinical manifestations [8, 9]. Currently, sepsis screening utilizes a variety of clinical variables and tools, such as Systemic Inflammatory Response Syndrome (SIRS) criteria, vital signs, signs of infection, Sequential Organ Failure Score (SOFA), Acute Physiology and Chronic Health Exercise Score II (APACHE-II), as well as biomarkers of inflammation and immunosuppression, to predict disease and mortality [10, 11]. Biomarkers have value in the diagnosis of infection, prognosis and treatment guidance of sepsis patients, which can help to provide reference for clinical decision-making and improve the prognosis of patient regression. Therefore, early recognition of sepsis, timely detection of disease progression and rapid intervention are of great significance in improving the prognosis and quality of life of patients.

Pitti et al. isolated a previously unknown full-length complementary DNA sequence for the first time from human embryonic lungs and named the protein it encodes decoy receptor 3 (DcR3) [12]. DcR3 is a 300 amino acid-containing polypeptide that is structurally similar to members of the tumor necrosis factor receptor (TNFR) family: the amino-terminal end contains a preamble sequence, followed by four tandem cysteine-rich structural domains (CRD). We can find DcR3 in the lungs, brain and liver of the fetus, as well as in the spleen, colon and lungs of the adult. As a biomarker of autoimmune and inflammatory diseases [13], DcR3 is difficult to detect in most normal tissues due to its low expression, but its expression is significantly up-regulated in inflammatory responses following some bacterial infections or organismal injuries, and is especially pronounced at SIRS.

Numerous studies have shown that DcR3 serum concentrations are significantly elevated in inflammatory bowel disease [13], rheumatoid immune system diseases [14], renal diseases, and acute respiratory distress syndrome (ARDS) [15], and independently predict 28-day mortality in ARDS patients [16]. Meanwhile, DcR3 is also highly expressed in many malignant tumors, especially pancreatic cancer [12], primary hepatocellular carcinoma, gastrointestinal malignancies [17], prostate cancer [18], and viral-associated lymphoma [19] and other oncological diseases. The high expression level of DcR3 in plasma is significantly correlated with the development of multiple organ dysfunction, and it is easy to see that serum DcR3 is a more valuable marker for predicting the outcome of inflammatory diseases.

In consequence, we aimed to perform this meta-analysis to synthesize the current evidence and investigate the diagnostic value of DcR3 on sepsis.

## Materials and methods

### Data sources and search strategy

We constructed a protocol of complete meta-analysis that adhered to the Preferred Reporting Items for Systematic Reviews and Meta-Analyses (PRISMA) standards [20]. The protocol was registered with the PROSPERO database (registration number CRD42023444411).

The investigators searched MEDLINE, Embase, Web of Science, Cochrane Library, China National Knowledge Infrastructure (CNKI) and Wanfang using the search term “ (“sepsis” OR “systemic inflammatory response syndrome”) AND (“receptors, tumor necrosis factor, member 6b” OR “decoy receptor 3” OR “DcR3”)”. For specific search details, see the Supplementary Material.

Two investigators independently searched the databases from the establishment of these databases until 7 January 2025 and reran the search before the final analysis. The screening process included assessing the articles’ titles, abstracts, and full text for eligibility and selecting relevant studies for synthesis that met the inclusion criteria. The research was done without language or publication period restrictions, and unpublished studies were not searched.

### Study selection criteria

The selected studies included the diagnostic value of DcR3 for differentiating sepsis patients, SIRS patients, and normal subjects. Sepsis and SIRS were diagnosed according to the recognized diagnostic criteria at the time each study was conducted, and there was no restriction on the type of study design eligible for inclusion. All retrieved articles were initially screened by title and abstract. Subsequently, relevant articles were rescreened through the full text.

Exclusion criteria: Studies that are not original clinical research, such as reviews, meta-analyses, case reports and animal studies; studies for which the full text is unavailable or key data are missing, making it impossible to pool effect sizes; duplicate publications of the same study, in which case only the most recent or most complete publication will be retained.

Screening was done independently by two researchers. They resorted to a third party in case of any disagreement during the study selection process. Decisions were recorded using EndNote.

### Data extraction

Two investigators independently extracted data from each report, including first author, year of publication, study site, study population, admission category (surgical or medical), and sample size. We also recorded true positives (TP), false positives (FP), false negatives (FN), true negatives (TN), sensitivity, specificity, area under the curve (AUC), and the optimal cutoff values for DcR3 to distinguish sepsis from normal and sepsis from SIRS. In case of disagreement between the two investigators during data extraction, it was resolved by a third party. If further information is required, the corresponding author will be contacted. If no response was received after a reminder was sent, the study was excluded. Extracted data were recorded in an Excel spreadsheet.

### Quality assessment

The quality assessment was done by two independent investigators using the Quality Assessment of Diagnostic Accuracy Studies 2 (QUADAS-2) tool [21]. This evaluation includes the following domains: patient selection, index test, reference standard, and flow and timing.

### Statistical analysis

Heterogeneity was assessed using Stata 13.0 software analysis with Q-test, *I²*-test and Galbraith plot. *I²* >50% with *P* < 0.05, was considered as the presence of heterogeneity and then analyzed by random effects model. Deeks funnel plot analysis was used to analyze the publication bias and *P* < 0.05 was considered as the presence of statistical heterogeneity. The sensitivity receiver operating characteristic curve (SROC) was fitted, and the combined sensitivity, specificity, positive likelihood ratio, negative likelihood ratio, and diagnostic odds ratio (DOR) were statistically analyzed by the random-effects model, and the AUC was calculated. Fagan nomogram visualization graphs, corrected for a priori probabilities to obtain posterior probabilities. Subgroup analyses were not conducted due to the small number of literatures screened for this study.

## Results

### Studies retrieved and their characteristics

**Fig. 1:**
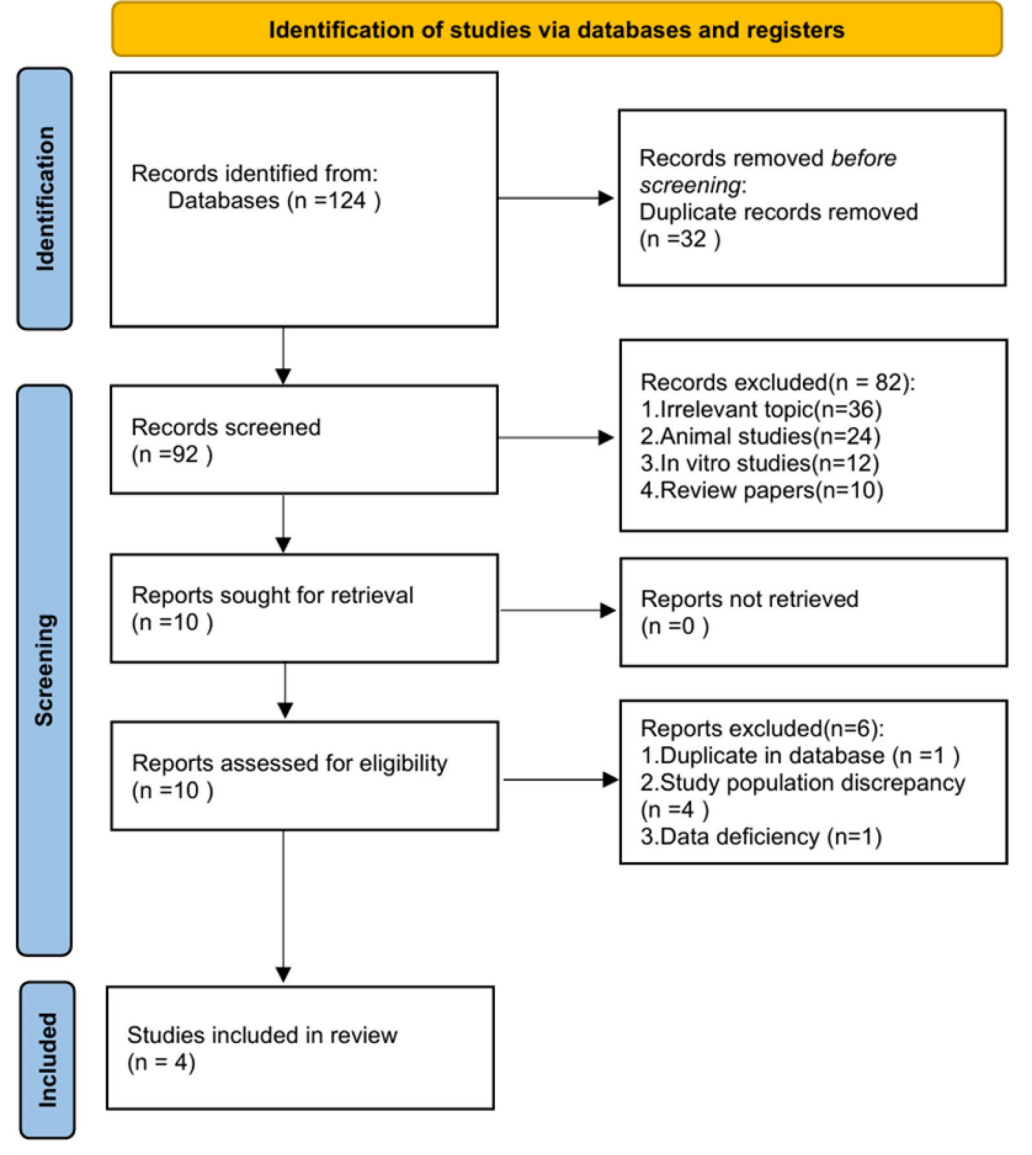
Preferred Reporting Items for Systematic Reviews and Meta-Analyses (PRISMA) flow diagram of the study selection

A total of 124 articles were obtained, and 92 articles were obtained after screening out duplicates (*n* = 32). The titles and abstracts of the articles were read to exclude irrelevant topics, animal studies, in *vitro* studies, and review papers, lastly a total of articles were excluded (*n* = 82). The articles were screened out after the initial screening (*n* = 10). The full text of the articles was read to exclude duplicate in database, study population discrepancy, and data deficiency, lastly a total of articles were excluded (*n* = 6). Four articles were finally included in the analysis [22–25]. The process of literature screening is shown in Fig. 1.

Tables 1 and 2 list the characteristics of these studies. All eligible studies were published between 2012 and 2018, and three of the articles were done in China. All of these studies were conducted in intensive care units (ICUs), and blood samples were collected on admission to the ICU. One study tested specimens for plasma and the other three studies tested specimens for serum. The populations of these studies were all adults. These studies used American College of Chest Physicians/Society of Critical Care Medicine (ACCP/SCCM) definition of sepsis and SIRS. The cutoff for DcR3 concentrations varied among these studies.

**Table 1:** The characteristics (Normal vs. Sepsis) of the included studies

Author	Year	Country	Population	Study design	Admission category	Sample	Cutoff (ng/mL)	Sample size	Age(y)	Gender (M%)	AUC	TP	FP	FN	TN	Sensitivity%	Specificity%	
Gao L	2017	China	Adult	Cohort study	Surgical	plasma	0.50	184	NR	NR	0.989	131	1	3	49	97.69	98.04	
Hou YQ	2012	China	Adult	Case-control study	Surgical	serum	1.10	212	67.5	63.0	0.910	23	10	1	178	97.7	82.2	
Zhao JJ	2018	China	Adult	Case-control study	NR	serum	0.55	54	54.3	57.6	0.990	33	2	1	18	97.1	90.0	
Sunghee Kim	2012	USA	Adult	Case-control study	Surgical &Medical	serum	1.65	71	54.2	NR	0.999	25	1	0	45	100	97.8	

**Table 2:** The characteristics (SIRS vs. Sepsis) of the included studies

Author	Year	Country	Population	Study design	Admission category	Sample	Cutoff (ng/mL)	Sample size	Age(y)	Gender (M/F)	AUC	TP	FP	FN	TN	Sensitivity%	Specificity%	
Gao L	2017	China	Adult	Cohort study	Surgical	plasma	1.96	194	55.4	62.5	0.95	121	1	13	59	90.1	98.4	
Hou YQ	2012	China	Adult	Case-control study	Surgical	serum	2.85	67	62.3	58.8	0.896	23	14	1	29	95.8	67.4	
Zhao JJ	2018	China	Adult	Case-control study	NR	serum	1.69	68	55.8	59.8	0.892	31	6	3	28	91.2	82.4	
Sunghee Kim	2012	USA	Adult	Case-control study	Surgical &Medical	serum	3.24	48	56.4	NR	0.958	24	4	1	19	96.0	82.6	

### Quality assessment and publication bias

**Fig. 2:**
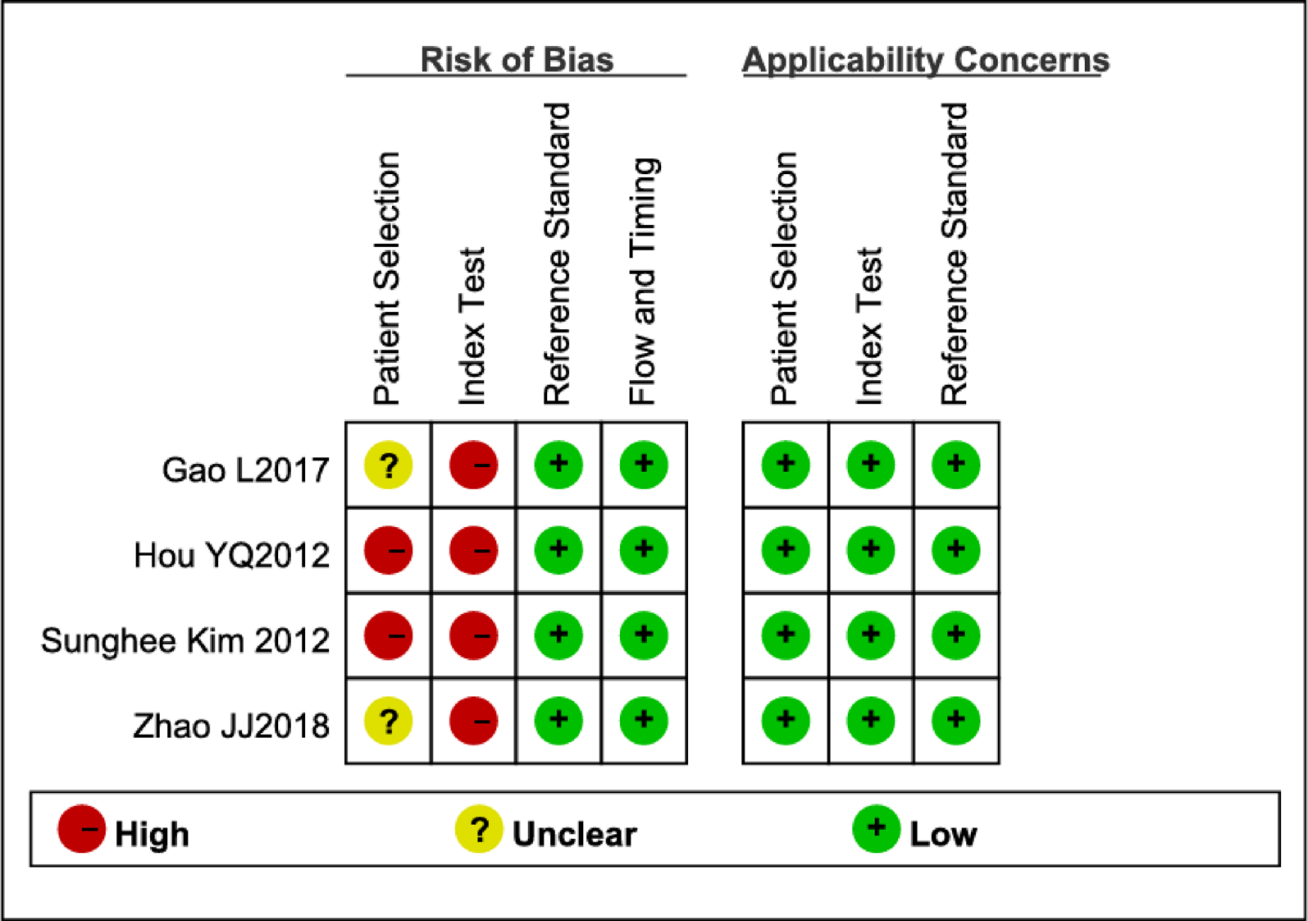
Risk of bias and applicability concerns of the included studies

The QUADAS-2 tool was applied to evaluate the quality of the four studies, and the detailed results were shown in Fig. 2.

### Data synthesis

**Fig. 3:**
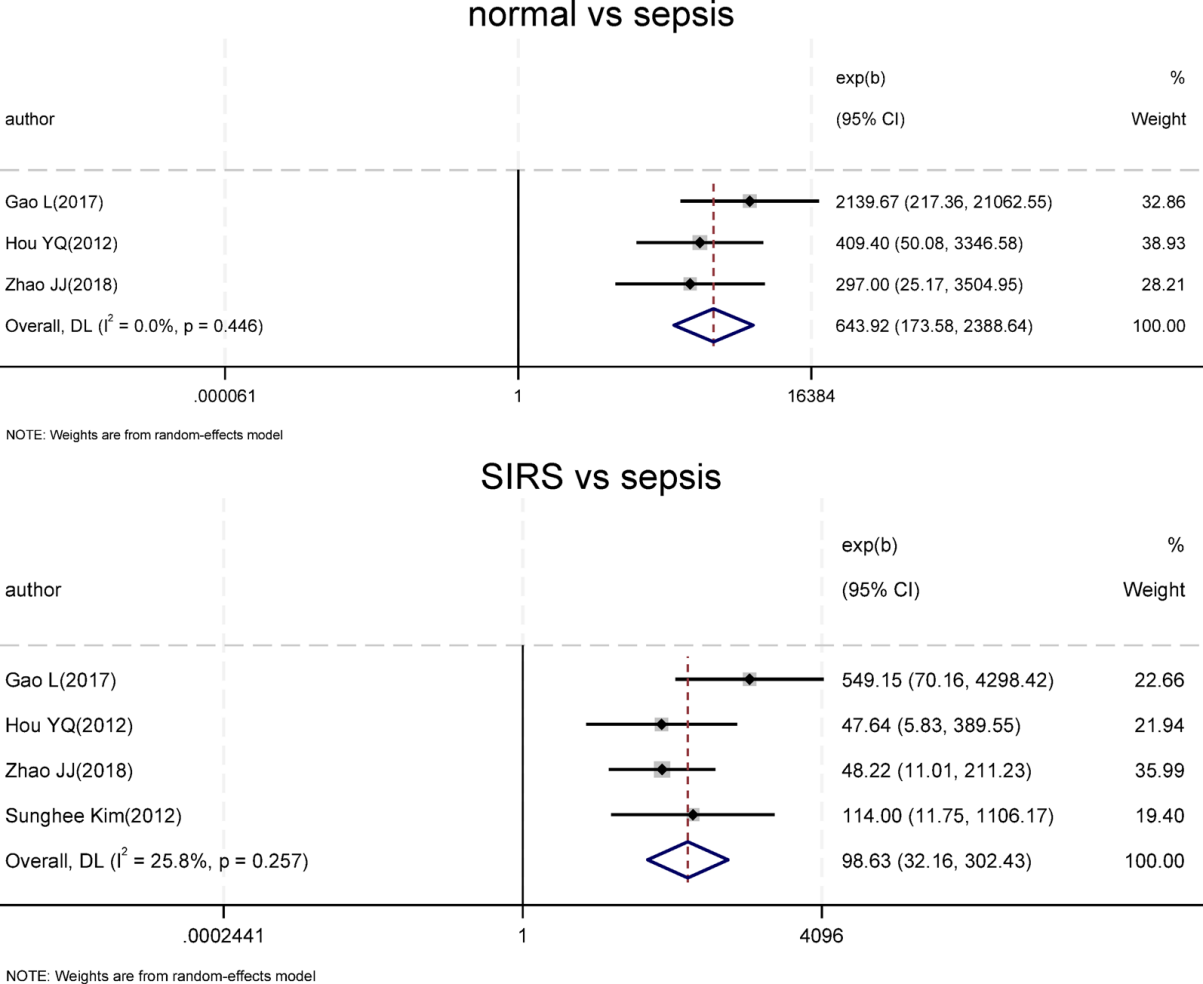
random-effects model (**a**: normal vs. sepsis; **b**: SIRS vs. sepsis)

By calculating Spearman’s correlation coefficients, Spearman’s rho=−0.6, *P* = 0.392>0.05 (normal vs. sepsis), and Spearman’s rho=−0.4, *P* = 0.591>0.05 (SIRS vs. sepsis), which indicated that there was no threshold effect in this study. In addition, the observed SROC curves showed that there was no “shoulder-arm shape”, further verifying that there was no threshold effect for the heterogeneity resulting from this study. Using the diagnostic ratio as the effect size, the Cochrane - Q test was 6.67, df = 3.00 (*P* = 0.08), I2 = 55.05 (normal vs. sepsis), and the Cochrane - Q test was 132.02, df = 3.00 (*P* = 0.00), I2 = 97.73 (SIRS vs. sepsis). The above indicated the presence of non-threshold induced heterogeneity, so the random-effects model was used (Fig. 3).

**Fig. 4:**
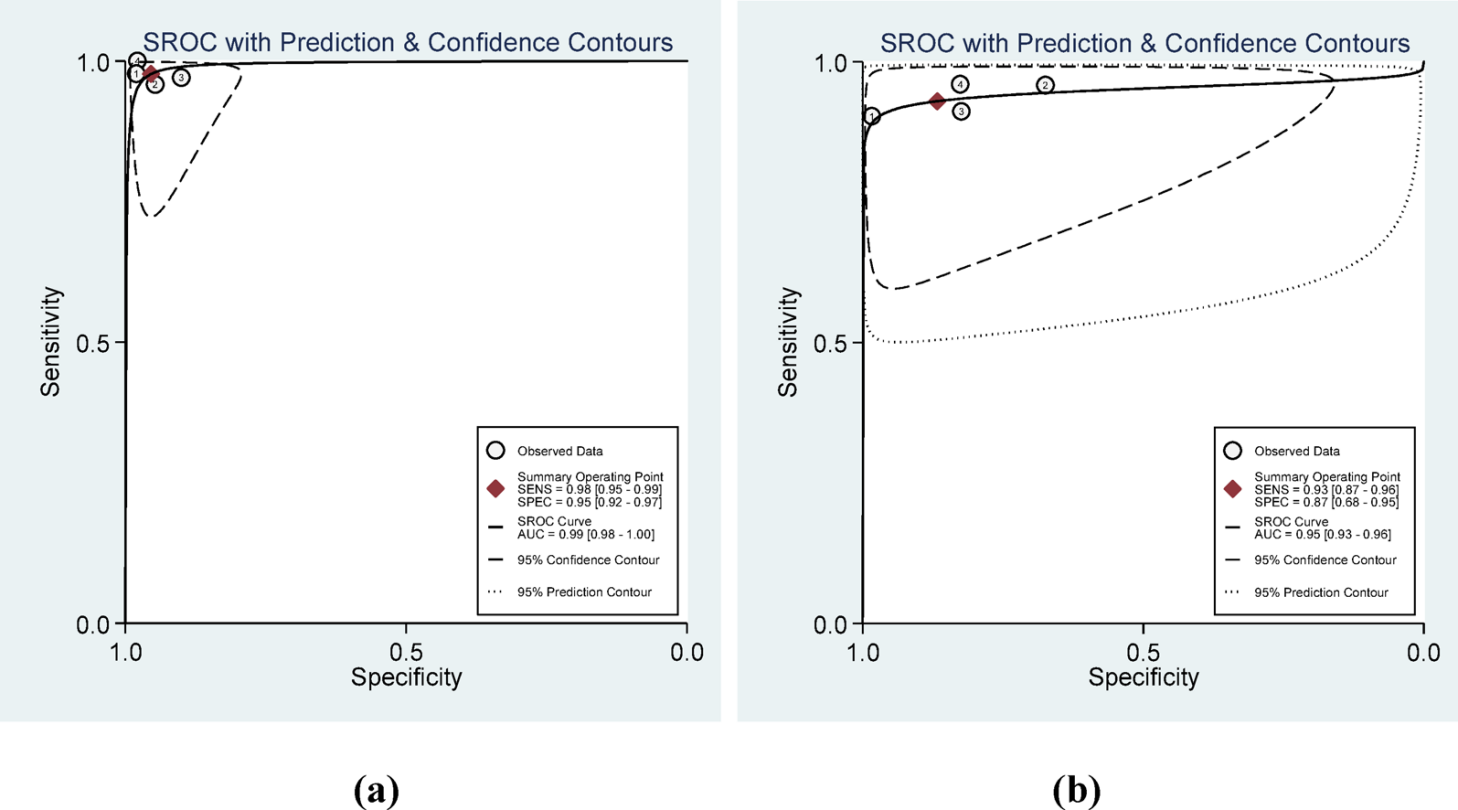
Summary receiver operating characteristic (SROC) curves for all included studies (**a**: normal vs. sepsis; **b**: SIRS vs. sepsis)

The SROC curve for all included studies is depicted in Fig. 4. Between normal and sepsis, the AUC was 0.99. The pooled sensitivity was 0.98 (95% CI 0.95–0.99), and the pooled specificity was 0.95 (95% CI 0.92–0.97). The pooled PLR was 21.21 (95% CI 12.71–35.40), the pooled NLR was 0.02 (95% CI 0.01–0.06), and the pooled DOR was 878.26 (95% CI 217.36–21062.55). Between SIRS and sepsis, the AUC was 0.95. The pooled sensitivity was 0.93 (95% CI 0.87–0.96), and the pooled specificity was 0.87 (95% CI 0.68–0.95). The pooled PLR was 6.89 (95% CI 2.67–18.26), the pooled NLR was 0.08 (95% CI 0.04–0.15), and the pooled DOR was 86.17 (95% CI 27.35–271.55). The HSROC model has adjusted for threshold effects, and the SROC curve is close to the upper left corner with a narrower confidence band, indicating a higher precision in the pooled estimate. Taken together, these results indicate that the results of this study are relatively stable.

**Fig. 5:**
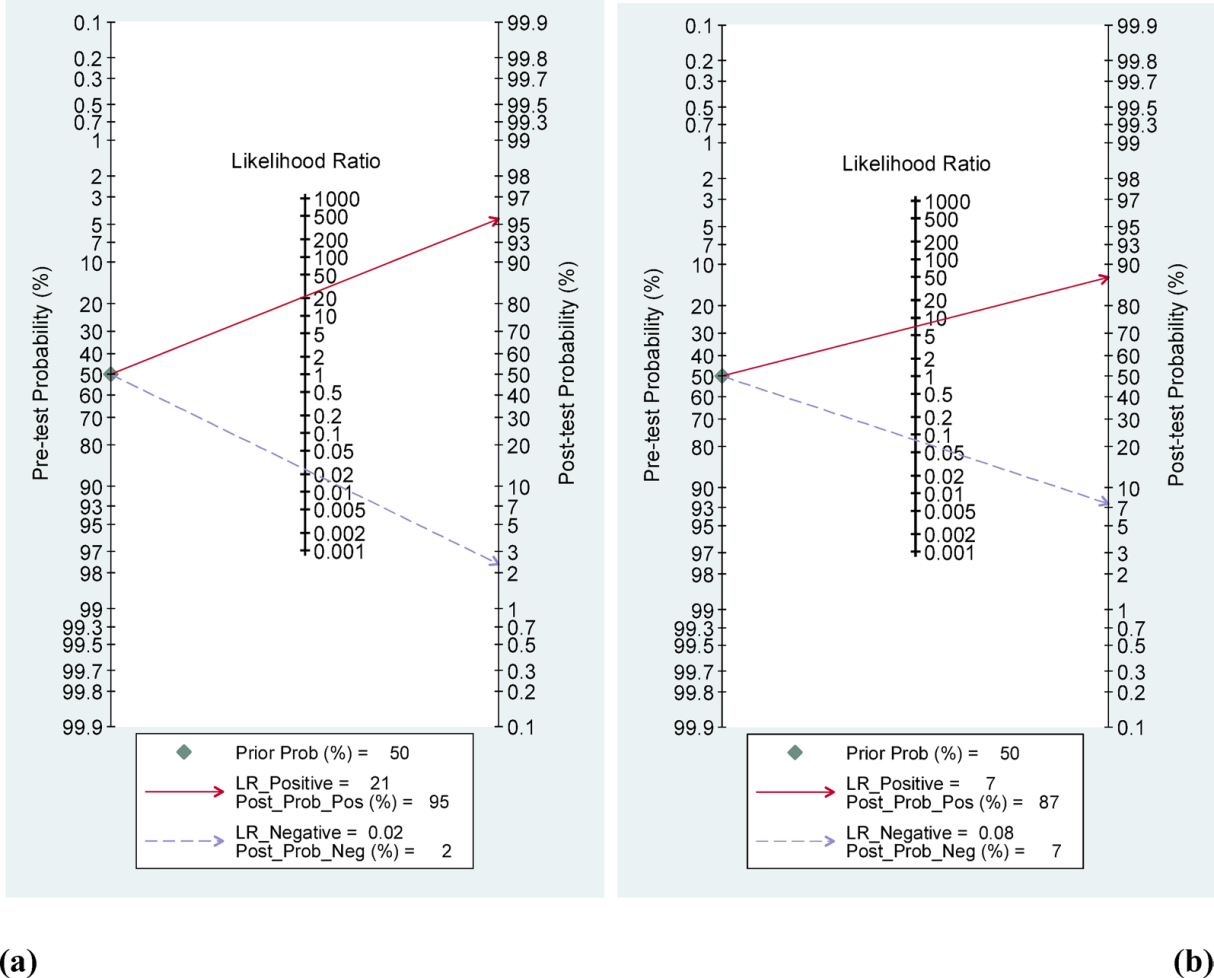
Fagan’s nomogram. (**a**: normal vs. sepsis; **b**: SIRS vs. sepsis)

Using a random-effects model, using a pre-test probability of 50% for the total adverse outcome. Between normal and sepsis, the post-test probability was 95%. Between SIRS and sepsis, the post-test probability was 87% (Fig. 5). It indicates that the application of DcR3 for early diagnosis of sepsis has a potential clinical application value.

### Sensitivity analysis and subgroup analysis

**Fig. 6:**
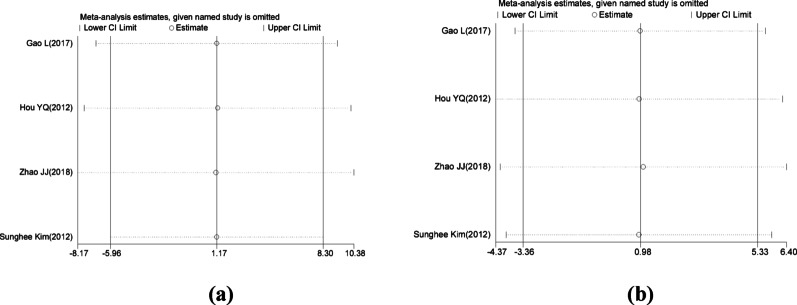
Sensitivity analysis. (**a**: normal vs. sepsis; **b**: SIRS vs. sepsis)

As can be seen from the Fig. 6, the sensitivity analysis was conducted using the case-by-case exclusion method, indicating that the results of the study were not affected because of individual studies and that the results of this study were relatively stable.

Regarding the included studies, the specimen type was plasma in one study and serum in three studies. The studies with sample sizes of more than 100 had two studies in normal vs. sepsis and one study in SIRS vs. sepsis. The results of subgroup analysis showed that sample size and specimen type maybe the source of heterogeneity, and the subgroup analysis is shown in Tables 3 and 4.

**Table 3:** Subgroup analysis (normal vs. sepsis)

Study	Subgroup	*n*	DOR	95%CI	I² (%)
Four studies		4	878.26	311.56–2475.75	55.05
Specimen type	plasma	1	2139.67	217.36–21062.55	0.00
	serum	3	359.00	98.14–1313.19	0.00
Sample size	> 100	2	877.75	174.45–4416.42	8.20
	≤ 100	2	331.11	63.69–1721.36	0.00

**Table 4:** Subgroup analysis (SIRS vs. sepsis)

Study	Subgroup	*n*	DOR	95%CI	I² (%)
Four studies		4	86.17	27.35–271.55	97.73
Specimen type	plasma	1	549.15	70.16–4298.42	0.00
	serum	3	58.11	19.99–168.90	0.00
Sample size	> 100	1	549.15	70.16–4298.42	0.00
	≤ 100	3	58.11	19.99–168.90	0.00

The study by Zhao JJ in 2018, due to its unique specimen type (plasma), possibly small sample size, and higher risk of bias in the areas of “patient selection” and “process and timing” according to the QUADAS-2(Fig. 2), became a key study contributing to the heterogeneity in the meta-analysis.

### Publication bias

**Fig. 7:**
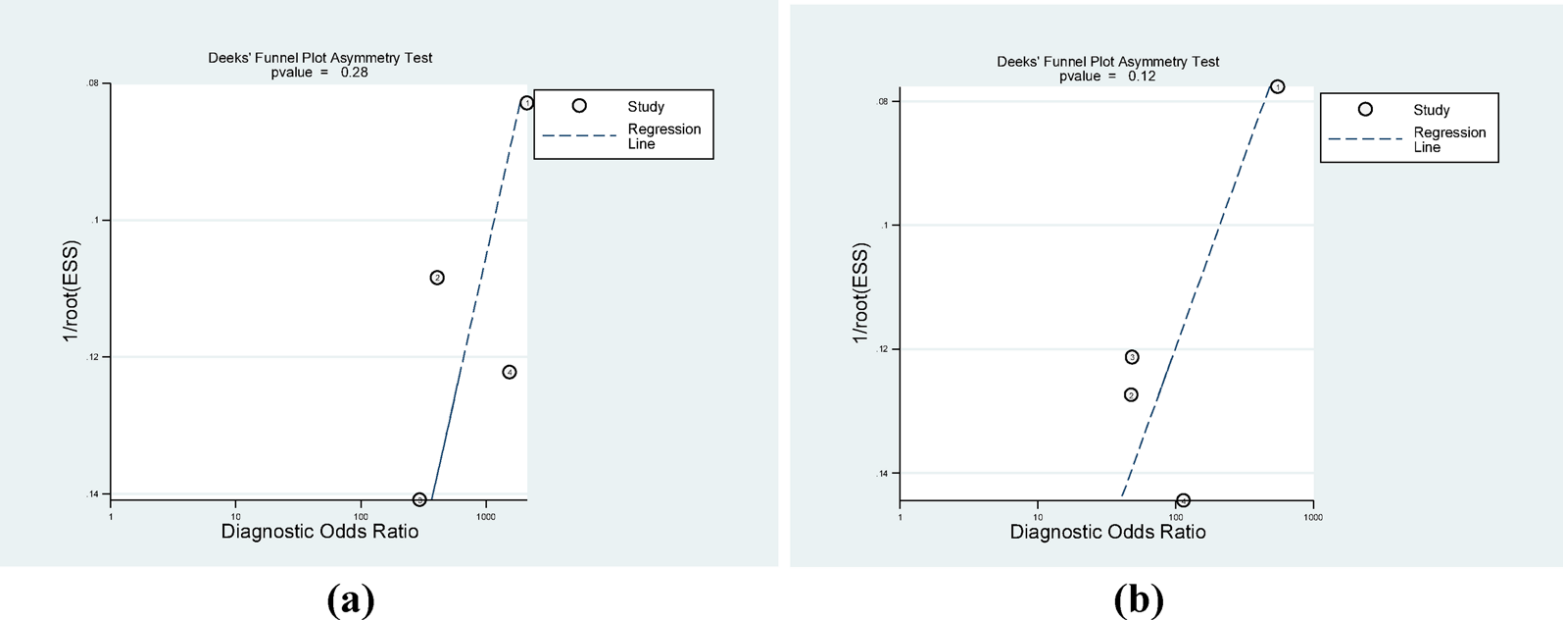
Deeks’ funnel plot. (**a**: normal vs. sepsis; **b**: SIRS vs. sepsis)

Deeks’ funnel plot asymmetry test (Fig. 7) results indicated that no significant publication bias existed between normal and sepsis, and between SIRS and sepsis in this meta-analysis (*P* = 0.28、*P* = 0.12). Due to the small number of studies, the statistical power of the Deeks test was limited, and its ability to assess publication bias was insufficient. Combined with a visual inspection of the scatter plot, no obvious asymmetry was observed, and it cannot yet be concluded that significant publication bias exists. However, given the small number of included studies, this conclusion should be interpreted with caution. Therefore, publication bias cannot be meaningfully ruled out based on this analysis alone. The possibility of unpublished negative studies that could affect the pooled estimates remains a concern.

### Heterogeneity analysis

**Fig. 8:**
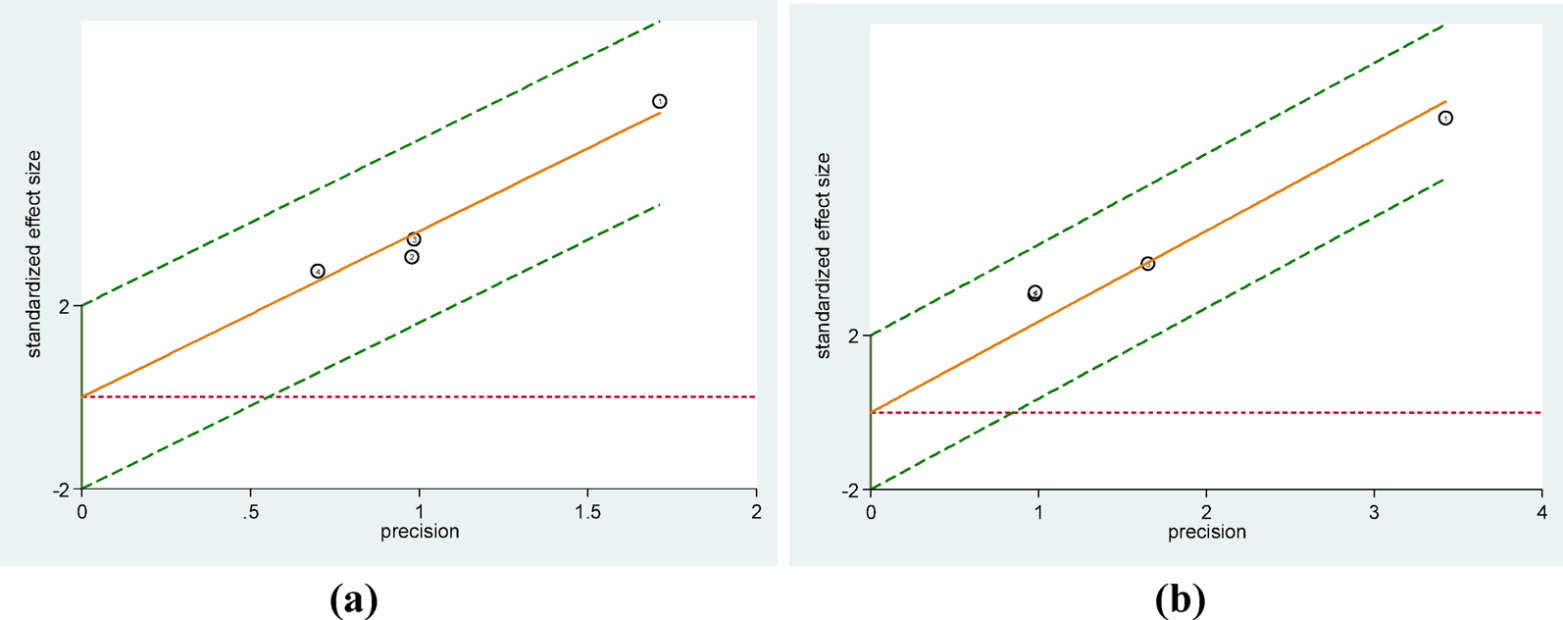
Galbraith plots. (**a**: normal vs. sepsis; **b**: SIRS vs. sepsis)

The Galbraith plot is suitable for analysing heterogeneity in studies with a small number of included studies. Each point on the plot represents a single study. The solid line in the centre represents the pooled effect size, whilst the dotted lines on either side represent the 95% confidence intervals. If a point lies outside the outer boundary of the dotted lines, that study is a major source of heterogeneity; if all points are largely within the dotted lines, this indicates that there are no sources of heterogeneity or that the heterogeneity is not significant. The Galbraith plot in this study shows that the distribution of the studies is relatively concentrated, and no obvious sources of heterogeneity were identified (Fig. 8).

## Discussion

In view of the morbidity and mortality of sepsis [26, 27], the diagnosis and treatment of sepsis has been a hot topic of research in the fields of emergency medicine, critical care medicine, infectious diseases, and even surgery since 2001. Sepsis experts have successively proposed versions 1.0, 2.0, and 3.0 of sepsis diagnosis, as well as concepts such as “Operation Save Sepsis”, “Early Goal-Directed Therapy (EGDT)”, “Intensive Treatment”, and methods such as “Restrictive Ventilation”, “Optimal Positive End-Expiratory Pressure (PEEP)”, and “Protective Pulmonary Ventilation”. However, after 20 years, the morbidity and mortality of sepsis have not been significantly reduced. Microbiological culture is the gold standard for the diagnosis of sepsis, but it is time-consuming and sometimes some negative culture results may not exclude the presence of pathogenic pathogens. Therefore, the current diagnosis and treatment of patients with sepsis is based on clinical and laboratory data of relatively poor accuracy. Currently, biomarkers of sepsis that are commonly used in clinical practice include CRP, IL-6, and PCT. However, these markers are not specific enough to distinguish sepsis from SIRS. Therefore, there is an urgent need for a better marker that can be used in the diagnosis of sepsis and to differentiate sepsis from SIRS.

An elevated PCT alone does not necessarily indicate a bacterial infection. Our body’s intrinsic immune system recognizes specific molecular fragments carried by pathogens through pattern recognition receptors (PRRs) on the surface of immune cells, i.e., pathogens-associated molecular patterns (PAMPs), like the pairing of a key with a lock, thus recognizing the pathogen. Through the signaling of immune cells, they release pro-inflammatory factors (TNF-a, IL-6, IL-8, etc.), which cause systemic inflammatory response and lead to elevated PCT. Therefore, bacterial PAMPs are the key to the initiation of inflammation and the elevation of PCT. Mitochondria are the only organelles possessing DNA other than the nucleus, and have a high degree of genetic similarity with bacteria. After cell necrosis, mitochondria rupture, releasing substances very similar to PAMPs, called damage-associated molecular patterns (DAMPs), which can also be recognized by PRRs, causing a similar These DAMPs can also be recognized by PRRs, causing similar severe infections or sepsis, which can also lead to elevated PCT. Therefore, non-infectious inflammatory reactions, such as severe trauma, major surgery, and burns, can lead to the release of a large number of DAMPs from the organism, resulting in the elevation of PCT. In conclusion, both PAMPs (infectious factors) and DAMPs (non-infectious traumatic factors) can cause SIRS and PCT elevation [28]. Moreover, PCT expression levels vary with the severity of the disease and the type of pathogens in the patients studied, and its efficacy in differentiating between sepsis and SIRS is unreliable [29].

In recent years, more and more studies have demonstrated that DcR3 plays a unique role in inhibiting inflammatory response and lymphocyte apoptosis [30]. DcR3 is not expressed or is poorly expressed under normal physiological conditions, but is highly expressed in patients with sepsis. Following bacterial or fungal infection, invading pathogens are captured, processed and presented by antigen-presenting cells (APCs) such as macrophages and dendritic cells (DCs). PAMPs such as lipopolysaccharide (LPS), phospholipid wall acid (LTA), and yeast glycan can bind to toll-like receptor 2/4 (TLR2/4) on the cell membrane, which activates downstream signaling pathways, such as nuclear factor-κB (NF-κB) and phosphatidylinositol 3-kinase protein kinase B (PI3K-AKT), and promotes DcR3 expression-associated gene transcription, leading to increased DcR3 content [31]. Many studies have demonstrated that DcR3 is specifically elevated in the serum of patients with sepsis, and may play a role in the differentiation and diagnosis of sepsis from SIRS [22–25]. It may play a role in the differential diagnosis between sepsis and SIRS [32].

A recent meta-analysis from 2025 [33] confirms that the PCT has limited diagnostic capability for sepsis with moderate discriminatory power (AUC 0.74, 95% CI: 0.62–0.84), and CRP is similar (AUC 0.67, 95% CI: 0.56–0.77)33. Based on existing studies and our meta-analysis, DcR3 demonstrates excellent diagnostic performance, and its ability to distinguish sepsis from SIRS or healthy individuals (high AUC, high sensitivity and specificity) is significantly superior to the performance of PCT/CRP revealed in the current meta-analysis.

Our results indicated that DcR3 showed prominent AUC and DOR in diagnosing sepsis and differentiating sepsis from SIRS. However, several limitations must be acknowledged.

When distinguishing between healthy individuals and sepsis patients, DcR3 has a high estimated DOR value (878.26), suggesting it has excellent discriminative potential. However, the 95% confidence interval of this estimate is abnormally wide (217.36–21062.55), indicating that the current pooled estimate of the DOR is extremely unstable and has very low precision. This is mainly due to the limited number of included studies (*n* = 4), heterogeneity between studies (I²=55.05%), and some individual studies contributing extreme and imprecise effect values (e.g., Gao L 2017). Consequently, while the point estimate suggests a strong diagnostic potential for DcR3, the current evidence base does not permit any definitive conclusions regarding the magnitude of its diagnostic performance. Any clinical interpretation of these findings must be approached with extreme caution, and the results should be considered preliminary until validated by larger, more methodologically robust studies.

In addition, subgroup analysis suggests that the main sources of heterogeneity may be the sample size and specimen type. However, due to the low number of articles included this time, the small sample size after subgroup division, the heterogeneity was magnified, so the correlation was high. Therefore, further studies are necessary to provide stronger evidence to support its reliable application in diagnosing sepsis and with differentiating sepsis from SIRS.

DcR3 levels are elevated to varying degrees in patients with SIRS and sepsis; the underlying cause lies in the fact that its expression is regulated by common core inflammatory signalling pathways (such as NF-κB). It represents a universal response to severe tissue damage and stress, playing a role in endogenous immune regulation and cell protection. In sepsis: After pathogens invade, pathogen-associated molecular patterns on their surfaces are recognized by Toll-like receptors on immune cells. Lipopolysaccharides from Gram-negative bacteria can activate the downstream NF-κB pathway through TLR4, while lipoteichoic acids from Gram-positive bacteria can do so through TLR2, thereby initiating the transcription of the DcR3 gene and leading to its significant upregulation. In SIRS: There is a large amount of cell necrosis. Following cell death, mitochondria rupture and release substances very similar to PAMPs. These DAMPs can also activate NF-κB through pattern recognition receptors such as TLR2/4, thereby inducing DcR3 expression in the same way. Consequently, its specificity (0.87) in distinguishing between infectious and non-infectious systemic inflammation is lower than its specificity (0.95) in distinguishing between healthy and diseased states. In conclusion, DcR3 is a sensitive response to widespread inflammatory damage, whether infectious or non-infectious, rather than a specific marker for sepsis. This directly explains why the DcR3 levels in some SIRS patients (especially those with severe conditions) significantly increase, overlapping with the levels in sepsis patients, which may lead to false-positive results. This characteristic suggests that DcR3 may need to be used in combination with other more infection-specific indicators such as PCT to improve the accuracy of differential diagnosis, rather than as a single specific indicator capable of absolutely distinguishing SIRS from sepsis.

Although our study has been strictly in accordance with the development of the inclusion and exclusion criteria for screening one by one, but the existence of the problem should not be underestimated.

The most commonly used diagnostic criteria for sepsis at present is Sepsis-3, yet the included studies used ACCP/SCCM criteria. It may also, to some extent, reduce the relevance and generalisability of the findings of this meta-analysis for current clinical practice; this is an important consideration when interpreting the conclusions of this study. Additionally, most included studies are from China, which may limit external validity, the generalizability of these findings to other ethnic or geographic populations remains uncertain. In addition, the sample types varied across the studies we included; one used plasma, whilst the others used serum.

The main value of our meta-analysis lies in systematically summarizing early evidence and pointing out clear research directions, rather than providing definitive final conclusions. The small sample size directly limits the reliability of the conclusions. While our Deeks’ test results were not statistically significant, this finding should not be interpreted as evidence of absence of publication bias. To obtain higher-level evidence (such as moderate or high certainty), it is necessary to conduct: large-sample, multicenter, multinational prospective diagnostic accuracy studies to provide more precise effect size estimates; more high-quality studies to enable more convincing subgroup analyses and publication bias assessments; direct comparative studies to compare DcR3 with existing standards (such as PCT) in the same population.

## Conclusion

In conclusion, DcR3 has a certain clinical reference value in diagnosing sepsis and differentiating SIRS from sepsis. However, previous clinical trial data from relevant studies are scarce and more larger, multi-center, non-case-control validation studies are needed to confirm this preliminary finding. Therefore, Until such evidence is available, DcR3 should not be considered a validated clinical diagnostic tool for sepsis.

### Take-home message

This study demonstrates the role of DcR3 in diagnosing sepsis and differentiating sepsis from SIRS. This study makes a valuable contribution to the field of diagnosis of sepsis by integrating and analyzing multiple clinical studies.

## Supplementary Information

Supplementary Material 1

## Data Availability

The original contributions presented in the study are included in the article. Further inquiries can be directed to the corresponding author.

## Abbreviations

DcR3: Decoy receptor 3
SIRS: Systemic inflammatory response syndrome
PLR: Positive likelihood ratio
NLR: Negative likelihood ratio
DOR: Diagnostic odds ratio
SROC: Summary receiver operator curve
AUC: Area under curve
