# Supplementary material for: Decoy receptor 3 as a diagnostic marker for sepsis: a meta-analysis
Source: Intensive Care Med Exp. 2026 Jul 2;14:87. doi: 10.1186/s40635-026-00943-z (PMC13328630; doi:10.1186/s40635-026-00943-z)
Supplement: Supplementary file 1 — Supplementary Material 1 [file 40635_2026_943_MOESM1_ESM.docx]

**Supplementary**

**Search Strategy**

1. PubMed

| Search number | Query | Search Details | Results |
| --- | --- | --- | --- |
| #3 | #1 AND #2 | ("sepsis"[MeSH Terms] OR "systemic inflammatory response syndrome"[MeSH Terms] OR "systemic inflammatory response syndrome"[MeSH Terms] OR "sepsis"[Title/Abstract]) AND ("receptors, tumor necrosis factor, member 6b"[MeSH Terms] OR "decoy receptor 3"[Title/Abstract] OR "DcR3"[Title/Abstract]) | 14 |
| #2 | ((dcr3 receptor[MeSH Terms]) OR (decoy receptor 3[Title/Abstract])) OR (DcR3[Title/Abstract]) | "receptors, tumor necrosis factor, member 6b"[MeSH Terms] OR "decoy receptor 3"[Title/Abstract] OR "DcR3"[Title/Abstract] | 360 |
| #1 | (((sepsis[MeSH Terms]) OR (sepsis syndrome[MeSH Terms])) OR (sepsis syndromes[MeSH Terms])) OR (sepsis[Title/Abstract]) | "sepsis"[MeSH Terms] OR "systemic inflammatory response syndrome"[MeSH Terms] OR "systemic inflammatory response syndrome"[MeSH Terms] OR "sepsis"[Title/Abstract] | 234,227 |

1. Embase

| Search number | Query | Results |
| --- | --- | --- |
| #3 | #1 AND #2 | 17 |
| #2 | 'decoy receptor 3'/exp OR 'decoy receptor 3' OR 'dcr3' | 631 |
| #1 | 'sepsis'/exp OR 'sepsis' | 426779 |

1. Web of Science

| Search number | Query | Results |
| --- | --- | --- |
| #1 | ((TS=(decoy receptor 3)) OR TS=(dcr3)) OR TS=(Receptors, Tumor Necrosis Factor, Member 6b) and Preprint Citation Index | 2914 |
| #2 | (TS=(sepsis)) OR TS=(sepsis syndrome) and Preprint Citation Index | 279081 |
| #3 | #1 AND #2 and Preprint Citation Index | 62 |
| #4 | #1 AND #2 | 62 |

1. Cochrane

| Search number | Query | Results |
| --- | --- | --- |
| #1 | MeSH descriptor: [Sepsis] explode all trees | 6663 |
| #2 | "sepsis syndrome" | 104 |
| #3 | #1 OR #2 | 6713 |
| #4 | MeSH descriptor: [Receptors, Tumor Necrosis Factor, Member 6b] explode all trees | 1 |
| #5 | "decoy receptor 3" | 2 |
| #6 | DcR3 | 2 |
| #7 | #4 or #5 or #6 | 2 |
| #8 | #3 and #7 | 0 |

1. CNKI

((SU=脓毒症 OR SU=脓毒血症)) AND ((SU=诱骗受体3 OR SU=DCR3 OR SU=DcR3 OR SU=肿瘤坏死因子6 OR SU=TNFR6))

1. Wanfang

(主题:(脓毒症) or 主题:(脓毒血症)) and (主题:("诱骗受体3") or 主题:("诱骗受体 3") or 主题:(DCR3) or 主题:(DcR3) or 主题:("肿瘤坏死因子6") or 主题:(TNFR6))
